# Knowledge, attitudes and practices of Zimbabwean men relating to prostate cancer

**DOI:** 10.4102/curationis.v46i1.2459

**Published:** 2023-11-13

**Authors:** Tendai Chisamba, Johanna E. Maree, Jacoba J. Jansen van Rensburg

**Affiliations:** 1 Department of Nursing Education, Faculty of Health Sciences, University of the Witwatersrand, Johannesburg, South Africa

**Keywords:** prostate cancer, knowledge, attitudes, practices, Harare, Zimbabwe

## Abstract

**Background:**

Prostate cancer is one of the leading causes of death in Zimbabwe. However, screening for prostate cancer is opportunistic as population-based screening is not available.

**Objectives:**

This study aimed to describe the knowledge, attitudes and practices of men living in Harare, Zimbabwe relating to prostate cancer.

**Method:**

A door-to-door survey took place in Mufakose, Harare. Each household was included, and men, 40 years and older, were convenience sampled until realisation of the calculated sample size of 269 (*n* = 269). A researcher-administered questionnaire collected the data, analysis was performed with descriptive statistics, and Chi-square tested statistically significant differences between the variables.

**Results:**

The majority of the sample (53.2; *n* = 143) was between 40 years old and 49 years old. Most (74.5%; *n* = 201) did not know what prostate cancer was, but the total sample (100%; *n* = 269) indicated that prostate cancer could lead to death. Only 50.6% (*n* = 136) were of the opinion that men should be screened; most (87.7%; *n* = 236) had never been screened. A Chi-square test of independence found a statistically significant difference between educational level and having had prostate cancer screening, χ^2^ (1) = 47.881, *p* < 0.000.

**Conclusion:**

As confirmed by other studies, the respondents had limited knowledge of prostate cancer, but had a positive attitude towards the disease, as most were willing to go for screening. There had been only a small percentage screened previously, and less than half returned to learn the results.

**Contribution:**

The study emphasises the role of primary health clinics as it could be an ideal setting to teach men about prostate cancer and its screening, and provide screening services in Zimbabwe.

## Introduction

Prostate cancer is a worldwide health problem and according to the 2020 Globocan statistics (Sung et al. 2021), it is the second most common cancer and the fifth most common cause of cancer deaths among men. In 2020, approximately 1.4 million men were newly diagnosed with prostate cancer, while about 3 75 000 died from this disease. Southern Africa is one of the regions with the highest incidence with sub-Saharan Africa being one of the regions with the highest mortality rates. In addition, there have been rapidly increasing trends, ranging from 2% to 10% per year, found in various sub-Saharan countries including Zimbabwe, over the period 1995–2018; the reason is unclear. Prostate cancer is the most common cancer in men in Zimbabwe, and the number of deaths recorded from it could be higher as it has been hampered by underreporting and a lack of accurate diagnosis (Chokunonga et al. 2013).

Despite the high incidence rates worldwide, there is little knowledge about the aetiology of prostate cancer except for established risk factors, such as advanced age, family history of prostate cancer, conditions such as Lynch Syndrome and certain genetic mutations influencing genes that produce proteins to help repair damaged DNA such as *BRCA1* and *BRCA2*. However, smoking, being overweight and having high androgen levels are associated with prostate cancer (Sung et al. 2021; Tadman, Roberts & Foulkes 2019).

Approximately 50% of men diagnosed with prostate cancer are asymptomatic and had their cancer detected by means of prostate specific antigen (PSA) screening. However, men can experience urinary symptoms, such as urinary frequency and retention, a weak stream and haematuria. Prostate cancer can spread directly to the adjacent organs, such as the rectum and bladder, and also to the regional lymph nodes and distant sites, usually bone (Tadman et al. 2019). In advanced stages, men may experience bone pain, often in the spine or pelvis, leg weakness, lymphoedema in the legs and genitals, urinary incontinence and weight loss. Factors such as prostate hypertrophy, embarrassment, a lack of knowledge, being asymptomatic and financial difficulties contribute to late presentation (So et al. 2014; Tadman et al. 2019).

Screening for prostate cancer is controversial and it appears to be shared decision making between men of 55 years and 69 years, and their physicians has replaced routine screening (Catalona 2018; Tan et al. 2019) in certain regions. However, screening aims at preventing and diagnosing prostate cancer at an early stage, which makes treatment easier and cure possible. The principles of screening of prostate cancer are measurement of serum PSA and a digital rectal examination (DRE) (Nakandi et al. 2013; Tadman et al. 2019). These tests are available in Zimbabwe, but, according to Makau-Barasa et al. (2022), are not commonly used for screening, rather opportunistically used for early diagnosis.

Knowledge of cancer, recognising the presenting symptoms and seeking healthcare when such symptoms are experienced, are key to the outcomes for the person. Similar to the rest of Africa, people living in Zimbabwe present with advanced cancer because of various reasons, including a lack of knowledge (Cassim et al. 2021; Nyakabau 2014) and fear of having a cancer diagnosis as a result of the lack of affordable treatment opportunities (Ngwa et al. 2022). Zimbabwe faces various challenges in terms of cancer prevention, detection and treatment, and increasing the public’s awareness of the signs and symptoms of cancer can facilitate early diagnosis (Nyakabau 2014). In addition, insight into what men know and practise in relation to prostate cancer can assist with the development of context-specific interventions to prevent and detect the disease at an early stage. Therefore, this study described the knowledge, attitudes and practices of men living in Zimbabwe relating to prostate cancer and prostate cancer screening. This information could guide nurses and other healthcare professionals and healthcare educators in planning preventative interventions.

## Methods

### Design

A door-to-door survey was conducted. This design was selected as, according to Hillier et al. (2014), door-to-door surveys are valuable tools while collecting information about health in urban areas.

### Study setting

The study setting was Mufakose, an urban, high-density suburb in the western part of Harare Central Business District, about 15 km from the city centre, and one of the oldest localities in Harare, initially designated for housing black Africans. Mufakose is a well-developed area, having electricity, piped water into all houses and modern sanitation. Mufakose comprises people from different cultural backgrounds who speak different languages, such as Shona, Ndebele, Zezuru, Tonga and English, the most common being Shona and English.

### Target population, sample and sampling method

The target population, as per the study’s requirements (Gray, Grove & Sutherland 2016), consisted of all men 40 years and older, living in Mufakose. The sample size was calculated using the Raosoft^®^ computer programme with a 5% margin error, confidence level of 90% and population of 52 921 (Luque Fernandez et al. 2012), resulting in a sample size of 270 (*n* = 270). Convenience sampling recruited the respondents who had to be 40 years and older and proficient in English or Shona for inclusion in the study.

### Data collection instrument and procedure

A self-developed questionnaire, based on the literature (Arafa, Farhat & Rabah 2015; Arnold-Reed et al. 2008; Ashford et al. 2001; Baaitse 2018; Matshela, Maree & Van Belkum 2014) and expert opinion, was used to collect the data. The questionnaire was researcher-administered and used a face-to-face approach. This approach has the advantages of including illiterate respondents and allowing the interviewer to assist respondents with issues that are not clear to them (ed. Maree 2016).

The questionnaire consisted of 28 questions, both open- and closed-ended, and was divided into four sections: demographic information, knowledge of prostate cancer, attitudes towards prostate cancer and practices pertaining to the disease. The questionnaire was pre-tested allowing the researcher to identify and refine errors specific to the population (Ikart 2019). Twenty-six (*n* = 26) eligible respondents were involved in the pre-test; there was no amendments to the questionnaire, and the pre-test data were not included in the results.

To reduce the cost of data collection, the first author collected the data after obtaining ethical clearance and site permission from the Harare City Council between January and February 2018. The researcher moved from one household to the next, and invited all men, who met the inclusion criteria, to complete the questionnaire after explaining the purpose of the study to them. Those who volunteered received an information leaflet, and after signing informed consent, had the questionnaire administered. Not all the eligible men were willing to participate; some demanded incentives and were excluded from the study. However, the sample realised at 269 (*n* = 269).

### Data analysis

The completed questionnaires went into envelopes and were numbered sequentially. The answers of the open-ended questions were analysed by means of content analyses (Gray et al. 2016), whereafter all the data were entered onto an Excel spreadsheet and analysed by means of the SPSS version 16 computer program using descriptive statistics. The Chi-square test of independence was used to determine relationships between the variables; critical value, *p* ˂ 0.05 (Gray et al. 2016).

### Ethical considerations

The study proposal was peer reviewed and the University of the Witwatersrand Human Research Ethics Committee (#M170566) and the Medical Research Council of Zimbabwe granted ethical clearance. To adhere to ethical principles, the researcher explained the study to all the recruited men, and those who volunteered received an information leaflet and signed informed consent whereafter the data were collected. No harm was intended and respondents had the right to withdraw from the study. By numbering the questionnaires, anonymity was maintained.

## Results

The ages of the respondents ranged from 40 to 81 years with a mean of 51.6 (standard deviation [s.d.] ± 8.9) and a mode of 49. The majority (53.2%; *n* = 143) was between the ages 40 and 49 years, members of the Zereru tribe (66.5%; *n* = 179), employed (70.6%; *n* = 190), and preferred to consult at primary health clinics (66.9%; *n* = 180). A quarter of the sample had no formal education (25.7%; *n* = 69). Table 1 presents the details.

**TABLE 1:** Demographic information (*N* = 269).

Variables	*n*	%
**Age (years)**		
40–49	143	53.2
50–59	70	26.0
60–69	41	15.2
70 >	15	5.6
**Tribe**		
Zezuru	179	66.5
Tonga	10	3.7
Karanga	20	7.4
Ndebele	50	18.6
Other	10	3.7
**Educational level**		
No formal education	69	25.7
Grade 1–7 (primary school)	61	22.7
Form 1–4 (secondary school)	59	21.9
Form 5–6 (advanced level secondary school)	64	23.8
Tertiary education	16	5.9
**Source of income**		
No personal income	50	18.6
Self-employed	100	37.1
Employed	90	33.5
Pension	29	10.8
**Preferred healthcare provider**		
Primary health clinics	180	66.9
Private doctors	36	13.4
Traditional healers	30	11.2
Spiritual healers	23	8.5

To investigate the sample’s knowledge of prostate cancer, the authors asked them if they knew what prostate cancer was, what they understood of the disease, whether they thought it could be prevented, what the risk factors were, which test could be performed to detect prostate cancer and how it is treated. On asking the respondents if they knew what prostate cancer was, only 24.2% (*n* = 65) responded positively; the rest either did not know (74.7%; *n* = 201) or were unsure (1.1%; *n* = 3). On asking what they understand about prostate cancer, the highest percentage (46.2%; *n* = 30) answered it is cancer of the ‘male parts’. To determine whether the sample knew what the signs of prostate cancer were, the respondents were asked what changes in their bodies would create suspicion of prostate cancer; nearly half (49.1%; *n* = 132) were of the opinion that newly onset of erectile dysfunction would be a sign. Most (59.1%; *n* = 159) did not know which screening tests could be used, and none were aware that hormonal therapy could treat this disease. Table 2 presents the details.

**TABLE 2:** Knowledge of prostate cancer (*N* = 269).

Question	*n*	%
**Do you know what prostate cancer is?**		
Yes	65	24.2
No	197	73.2
Unsure	7	2.6
**If yes, what do you understand prostate cancer is?**		
Cancer of the male parts	30	11.2
Cancer of the reproductive or urinary system	10	3.7
Cancer in men	9	3.3
Unsure	16	5.9
**Do you think prostate cancer can be prevented?**		
Yes	76	28.3
No	71	26.4
Unsure	122	45.3
**What would increase a man’s risk of developing prostate cancer?**		
Family history of cancer	52	19.3
Age	46	17.1
Consumption of red meat	11	4.1
Race	8	2.8
Other (smoking and drinking alcohol)	3	1.1
Unsure	149	55.4
**What would decrease a man’s risk of developing prostate cancer?**		
Balanced diet	7	2.6
Low fat diet	5	1.9
Reduced intake of red meat	0	0.0
Exercises	8	3.0
Screening	27	10.2
Other (specify) drinking African herbs	5	1.9
Unsure	217	45.3
**What changes in your body would make you think you might have prostate cancer?**		
Urinating frequently especially during the night	12	4.5
Delayed or prolonged urination	15	5.6
Weak urine stream	39	14.5
Incomplete emptying of the bladder	13	4.8
Pain when passing urine	2	0.8
Blood in urine	15	5.6
Change in bowel habits	36	13.4
New onset of erectile dysfunction	132	49.1
Unsure	5	1.9
**Which tests can check for the presence of prostate cancer?**		
Blood tests (PSA)	58	21.6
Digital rectal examination (DRE)	41	15.2
Biopsy	11	4.1
Unsure	159	59.1
**How is prostate cancer treated?**		
Surgery	52	41.3
Radiotherapy	20	15.9
Chemotherapy	54	42.8
Hormone therapy	0	0
Unsure	143	53.2

PSA, prostate specific antigen.

While cross tabulating ‘know what prostate cancer is’ and age, the authors found the highest percentage of men between the ages 40 and 49 years (26.6%; *n* = 37) indicated they knew what prostate cancer was. However, the Chi-square test of independence showed no statistically significant relationship between age and knowing what prostate cancer was, χ^2^ (1) = 2.908, *p* = 0.025. When cross tabulating educational level and ‘know what prostate cancer is’, the group with tertiary education represented the highest percentage (50%; *n* = 8). Respondents who attended secondary school forms 1 to 4, (18.6%; *n* = 11) were the least inclined to know about prostate cancer. A Chi-square test of independence showed no statistically significant relationship between education and knowing what prostate cancer was, χ^2^ (1) = 7.690, *p* = 0.104 (see Table 3).

**TABLE 3:** Knowing what prostate cancer was and age and educational level (*N* = 269).

Variable	Know what prostate cancer is		Do not know what prostate cancer is		Total		*p*
	*n*	%	*n*	%	*n*	%	
**Age (years)**	-	-	-	-	-	-	0.025
40–49	37	13.8	102	37.9	139	51.7	-
50–59	19	7.1	54	20.1	73	27.1	-
60–69	7	2.6	34	12.6	41	15.2	-
70 >	2	0.7	14	5.2	16	5.9	-
**Educational level**	-	-	-	-	-	-	0.104
No formal education	17	6.3	52	19.3	69	25.7	-
Grade 1–7	12	4.5	49	18.2	61	22.7	-
Form 1–4	11	4.1	48	17.8	59	21.9	-
Form 5–6	17	6.3	47	17.5	64	23.8	-
Tertiary education	8	3.0	8	3.0	16	5.9	-

When investigating attitudes, the respondents were asked whether they considered prostate cancer a dangerous disease, such as HIV, and whether prostate cancer could result in death. The majority (74.4%; *n* = 200) agreed that prostate cancer was as dangerous as HIV, and all the respondents (100%; *n* = 269) indicated that men who have prostate cancer can die from it. We also asked whether prostate cancer was treatable, who should go for screening, and whether it is important to have screening every 5 years. Table 4 provides the details.

**TABLE 4:** Attitudes towards prostate cancer (*N* = 269).

Question	*n*	%
**Do you see prostate cancer as a dangerous illness like HIV?**		
Yes	200	74.4
No	9	3.3
Unsure	60	22.3
**Do you think prostate cancer can kill a man?**		
Yes	269	100
No	0	0
Unsure	0	0
**Do you think prostate cancer is treatable?**		
Yes	136	50.6
No	13	4.8
Unsure	120	44.6
**Do you think all men above 40 years should go for screening?**		
Yes	136	50.6
No	28	10.4
Unsure	105	39.0
**Do you think only men with urinary problems should go for screening?**		
Yes	124	46.1
No	35	13.0
Unsure	110	40.9
**Do you think it is important to go for screening every 5 years?**		
Yes	186	69.1
No	9	3.3
Unsure	74	27.6

As seen in Table 4, there were six questions asked about attitudes. To judge whether the sample’s attitudes were positive or negative, the authors considered three and more correct answers as a positive attitude. The majority of respondents (69.5%; *n* = 187) had a positive attitude towards prostate cancer, while 30.5% (*n* = 82) had a negative attitude. The highest percentage of positive attitudes (70.5%; *n* = 98) came from the 40 year old to 49 year old group while the lowest percentage came from the group who had no formal education (50%; *n* = 8); Chi-Square did not find a statistically significant difference between age and attitudes, χ^2^ (1) = 3.883, *p* = 0.274. When cross tabulating positive attitudes and educational level, the group who completed secondary school forms 5 to 6, scored the highest (75%; *n* = 48), while the lowest percentage (33.3%; *n* = 23) came from the group with no formal education. There was no statistically significant difference between attitudes and educational level, χ^2^ (1) = 4.386, *p* = 0.236 (see Table 5).

**TABLE 5:** Attitudes towards prostate cancer and age and educational level (*N* = 269).

Variable	Positive attitude		Negative attitude		Total		*p*
	*n*	%	*n*	%	*n*	%	
**Age (years)**	-	-	-	-	-	-	0.274
40–49	98	36.4	41	15.2	139	51.7	-
50–59	54	20.1	19	7.1	73	27.1	-
60–69	27	10.0	14	5.2	41	15.2	-
70 >	8	3.0	8	3.0	16	5.9	-
**Educational level**	-	-	-	-	-	-	0.236
No formal education	46	17.1	23	8.6	69	25.7	-
Grade 1–7	40	14.9	21	7.8	61	22.7	-
Form 1–4	39	14.5	20	7.4	59	21.9	-
Form 5–6	48	17.8	16	5.9	64	23.8	-
Tertiary education	14	5.2	2	0.7	16	5.9	-

To investigate practices, the respondents were asked whether they had heard of prostate cancer screening, what tests were used, whether they would be prepared to be screened and the reasons for their answers. The authors also asked those who were screened where they had it performed, and if they returned to learn the results. The majority of the respondents (62.8%; *n* = 169) were not sure about having heard of screening and only 12.2% (*n* = 33) had ever been screened. The majority of those not screened (78.8%; *n* = 212) indicated they were willing to undergo screening primarily ‘to know my status’ (54.7%; *n* = 116). Those who were not willing to be screened (33.7%; *n* = 57) indicated ‘they were not ill’ as reason for not wanting screening (87.7%; *n* = 50). Those who had been screened were mostly screened by a private doctor (60.6%; *n* = 33), while 54.5% (*n* = 18) said they never returned for the results. Table 6 presents the details.

**TABLE 6:** Practices regarding prostate cancer screening (*N* = 269).

Question	*n*	%
**Have you ever heard about prostate cancer screening?**		
Yes	60	22.3
No	40	14.9
Unsure	169	62.8
**If yes, can you please tell me the test used? (*n* = 60)**		
Blood test	40	66.7
Rectal examination	17	28.3
Other	3	5.0
Unsure	0	0
**Would you be prepared to go for prostate cancer screening?**		
Yes	212	78.8
No	57	21.2
Unsure	0	0
**If yes, why? (*n* = 212)**		
To know my status	116	54.7
Age above 40 years	40	18.9
Family history	14	6.6
Routine check up	6	2.8
To get treatment	40	18.9
**If no, why? (*n* = 57)**		
Not sick	50	87.7
Not experiencing any symptoms	7	12.3
**Have you ever had prostate cancer screening?**		
Yes	33	12.3
No	236	87.7
Unsure	0	0
**If yes, where did you have prostate cancer screening? (*n* = 33)**		
Private doctor	20	60.6
Primary healthcare clinic	0	0
Government hospital	13	39.4
Other	0	0
**If yes, what test/procedure did you have? (*n* = 33)**		
Blood test (PSA)	22	66.7
Rectal examination	9	27.3
Biopsy	2	6
**If you had a screening test done, did you return to get the results?**		
Yes	15	45.5
No	18	54.5

PSA, prostate specific antigen.

When cross tabulating age and having heard about prostate cancer screening, the highest percentage who had heard about prostate cancer screening (10.1%; *n* = 14) was between the ages 40 and 49 years, followed by those between 50 and 59 years (15.1%; *n* = 11). Chi-square found no statistically significant difference between having heard of prostate cancer screening and age, χ^2^ (1) = 5.040, *p* = 0.169. When cross tabulating educational level and having heard about prostate cancer screening, the highest percentage (62.5%; *n* = 10) was those who had tertiary education. A statistically significant difference was found between educational level and having heard about prostate cancer screening according to Chi-square, χ^2^ (1) = 59.565, *p* = 0.000 (Table 7). When cross tabulating age and having been screened, the highest percentage who had been screened (13.7%; *n* = 19) was between the ages 40 and 49 years, and none of the respondents who were 70 years and older had been screened. No statistically significant difference was found between age and having had prostate cancer screening, χ^2^ (1) = 5.7509, *p* = 0.124. When cross tabulating educational level and having had prostate cancer screening, the highest percentage (62.5%; *n* = 10) who had been screened had a tertiary education. A statistically significant difference was found between educational level and having had prostate cancer screening according to Chi-square, χ^2^ (1) = 47.881, *p <* 0.000 (see Table 7).

**TABLE 7:** Have heard of prostate cancer screening and had been screened previously (*N* = 269).

Variable	Heard about prostate screening		Have not heard about prostate screening		Total		*p*	Previously screened		Not been screened previously		Total		*p*
	*n*	%	*n*	%	*n*	%		*n*	%	*n*	%	*n*	%	
**Age (years)**	-	-	-	-	-	-	0.169	-	-	-	-	-	-	0.124
40–49	14	5.2	125	46.5	139	53.7	-	19	7.1	120	44.6	139	51.7	-
50–59	11	4.1	62	23.0	73	28.2	-	12	4.5	61	22.7	73	27.1	-
60–69	2	0.7	39	14.5	41	15.8	-	2	0.7	39	14.5	41	15.2	-
70 >	0	0.0	16	5.9	16	6.2	-	0	0.0	16	5.9	16	5.9	-
**Educational level**	-	-	-	-	-	-	0.000	-	-	-	-	-	-	< 0.000
No formal	2	0.7	67	24.9	69	25.7	-	3	1.1	66	24.5	69	25.7	-
Grade 1–7	2	0.7	59	21.9	61	22.7	-	3	1.1	58	21.6	61	22.7	-
Form 1–4	3	1.1	56	20.8	59	21.9	-	5	1.9	54	20.1	59	21.9	-
Form 5–6	10	3.7	54	20.1	64	23.8	-	12	4.5	52	19.3	64	23.8	-
Tertiary	10	3.7	6	2.2	16	5.9	-	10	3.7	6	2.2	16	5.9	-

## Discussion

The study provided evidence that the respondents had limited knowledge of prostate cancer, as only 24.2% indicated they knew what prostate cancer was and more than a third of those who said they knew, did not display a reasonable understanding of the disease. A similar trend was found by Moyo (2017), who, in a study conducted in rural Zimbabwe, found a very low level of awareness and knowledge of prostate cancer marred by misconceptions. Nakandi et al. (2013), who studied a group of men in Uganda, support these findings. These authors found 45.9% of their sample (*n* = 545) had heard about prostate cancer, but when exploring their understanding, found many of the men thought it was gonorrhoea. In contrast, Osei Agyemang et al. (2022), in a study conducted in Ghana, found that 85.8% of their sample of 426 men were aware of prostate cancer, with 52.5% having had adequate knowledge.

Despite having limited knowledge of prostate cancer, it was interesting that all the respondents were of the opinion that prostate cancer could lead to death, and about three-quarters considered prostate cancer to be as dangerous as HIV – the major cause of death in Zimbabwe (Centers for Disease Control and Prevention 2019). Both Olaoye, Baderinwa and Oyerinde (2022) and Moyo (2017) support this finding, as the majority of the men in their studies indicated that prostate cancer was a deadly disease. However, Nakandi et al. (2013) found that, although the majority of their respondents considered prostate cancer to be as bad as HIV and/or AIDS, they were only concerned about having HIV testing.

The total sample could identify one sign of prostate cancer, and the highest percentage (49.1%) identified new onset of erectile dysfunction as a sign. Whether knowing one sign is having knowledge, is debatable; however, the mere fact that all the respondents were able to identify at least one sign was positive, as Matshela et al. (2014) and Nakandi et al. (2013) found that approximately half of their participants, could not identify a single sign of prostate cancer. Whether the men in this study really knew erectile dysfunction was a sign of prostate cancer, or derived it from the nature of the study focusing on ‘men’s problems’ is not clear, as Mofolo et al. (2015) and Olapade-Olaopa et al. (2014) in their studies, found difficulty in passing urine was the most well-known sign among the men. This study’s finding cannot be highlighted as unique until further investigations are carried out.

Knowledge about who should undergo screening for prostate cancer was also low, as less than 9.0% of the respondents mentioned men with prostate cancer and men with a family history of prostate cancer. Moyo (2017) also found that rural Zimbabwean men had little knowledge of who should have screening. In addition, more than 80% of the respondents in this study who were not willing to be screened, did not want to because they were not ill. This is in contrast with the finding of Mutua, Pertet and Otieno (2017) who found the majority of their sample of Kenyan men (66%) believed they were at risk of prostate cancer, while 44% intended to be screened in the 6 months following the study.

Knowledge of men regarding screening was equally low, as only 22.3% of the respondents indicated they had heard about prostate cancer screening. In addition, less were able to identify the screening tests. However, this low percentage compared favourably to the 9% reported by Nakandi et al. (2013) and the 3% found by Moyo (2017). Matshela et al. (2014) found a similar trend, as only 4.6% of their participants were able to identify the PSA and 1.5% the DRE before their educational intervention. Not knowing about prostate cancer screening poses a serious risk to African men, as it is the primary reason for delayed screening and diagnosis, late presentation and delayed treatment, resulting in poor patient outcomes, higher treatment costs and a higher mortality (Moyo 2017).

As seen from this study, only 12.3% of respondents had had screening. It seems as if this low figure represents the situation in various African countries. For instance, Nakandi et al. (2013), in their study conducted in Kenya, found only 2.8% of their sample had been screened previously. Olapade-Olaopa et al. (2014) reported better results, as 21.5% of their sample of Nigerian men (*n* = 656) had a DRE and 17.1% had undergone a PSA test. In contrast, Yeboah-Asiamah et al. (2017) found that only 10% of their sample of Ghanaian male teachers (*n* = 160) had been screened, while Kangmennaangm, Mkandawire and Luginaah (2016), in a Namibian study, found only 16% of the men in their study were screened.

Adding to the concern about the low screening uptake, the current study provided evidence that the majority of the men who had undergone screening did not receive the results. Matshela et al. (2014) found a similar trend, as 41.5% of their participants, did not return to the clinic to learn their results and were lost to follow up. The reason for this situation is not clear and guiding literature is sparse; however, Vidhubala et al. (2020) found financial issues, a lack of family support, fear of the disease, misunderstanding of the screening procedure and sociocultural beliefs were barriers to follow up after cervical cancer screening. Whether the same barriers apply to men in Africa needs investigation before making conclusions.

This study found a statistically significant relationship between having had prostate cancer screening and educational level. This is not unique, and it is supported by various studies. Baratedi et al. (2020), while synthesising the barriers to prostate cancer screening among men living in sub-Saharan Africa, found that men with secondary and higher education were more likely to be screened. In addition, there was a steady gradient observed according to educational levels, with men without formal education the least likely to undergo screening and those with tertiary education the most likely. Similar to the studies of Nakandi et al. (2013) and Mofolo et al. (2015), age did not seem to play a role in taking up screening, as this study did not find a significant relationship between having had screening and age.

It was positive to notice that the majority of this study’s sample (69.5%) showed a positive attitude towards prostate cancer, and that this positive attitude applied across all educational levels and all age groups. Furthermore, the majority of the respondents confirmed the importance of going for prostate cancer screening every 5 years. Ugochukwu et al. (2019) also found a positive attitude among Nigerian men towards screening; however, this, as also seen in this study, did not realise in practice.

## Strengths and limitations

This study investigated knowledge, attitudes and practices of men living in Harare, Zimbabwe relating to prostate cancer, the most common cancer in Zimbabwean men. However, the study had various limitations. The study was conducted in one high-density suburb in Harare, using a non-probability sampling method, and therefore the results may not be generalised. A questionnaire collects self-report data that could have led to recall bias and socially acceptable answers, especially when data of a sensitive nature are collected. Using a face-to-face approach could have led to interviewer bias; however, this limitation was mitigated by using a well-trained interviewer. In addition, a survey tends to reflect relatively superficial knowledge of the topic investigated.

## Conclusion

As confirmed by previous studies, the men included in this study had limited knowledge of prostate cancer but a positive attitude towards the disease, as most were willing to go for screening. Only a small percentage had had screening previously and less than half returned to learn the results. This situation complicates health-seeking behaviour, early diagnosis and treatment, and enhances poor patient outcomes. Primary health clinics, the preferred healthcare provider, could be the ideal setting to teach men about prostate cancer and its screening, and provide screening services in Zimbabwe.
